# Structure determination of a major facilitator peptide transporter: Inward facing PepT_St_ from *Streptococcus thermophilus* crystallized in space group P3_1_21

**DOI:** 10.1371/journal.pone.0173126

**Published:** 2017-03-06

**Authors:** Esben M. Quistgaard, Maria Martinez Molledo, Christian Löw

**Affiliations:** 1 Centre for Structural Systems Biology (CSSB), DESY and European Molecular Biology Laboratory Hamburg, Hamburg, Germany; 2 Department of Medical Biochemistry and Biophysics, Karolinska Institutet, Stockholm, Sweden; University of Bern, SWITZERLAND

## Abstract

Major facilitator superfamily (MFS) peptide transporters (typically referred to as PepT, POT or PTR transporters) mediate the uptake of di- and tripeptides, and so play an important dietary role in many organisms. In recent years, a better understanding of the molecular basis for this process has emerged, which is in large part due to a steep increase in structural information. Yet, the conformational transitions underlying the transport mechanism are still not fully understood, and additional data is therefore needed. Here we report in detail the detergent screening, crystallization, experimental MIRAS phasing, and refinement of the peptide transporter PepT_St_ from *Streptococcus thermophilus*. The space group is P3_1_21, and the protein is crystallized in a monomeric inward facing form. The binding site is likely to be somewhat occluded, as the lobe encompassing transmembrane helices 10 and 11 is markedly bent towards the central pore of the protein, but the extent of this potential occlusion could not be determined due to disorder at the apex of the lobe. Based on structural comparisons with the seven previously determined P2_1_2_1_2_1_ and C222_1_ structures of inward facing PepT_St_, the structural flexibility as well as the conformational changes mediating transition between the inward open and inward facing occluded states are discussed. In conclusion, this report improves our understanding of the structure and conformational cycle of PepT_St_, and can furthermore serve as a case study, which may aid in supporting future structure determinations of additional MFS transporters or other integral membrane proteins.

## Introduction

Cellular uptake of dietary di- and tripeptides represents an important means of acquiring nitrogen and amino acids in many organisms [1–4]. In bacteria, this task is carried out by both ATP-binding cassette (ABC) transporters and major facilitator superfamily (MFS) transporters of the peptide transporter (PepT or PTR) family, also commonly referred to as the proton-dependent oligopeptide transporter (POT) family [5]. In contrast, humans use only PepTs for this task [5], of which the two best studied are the intestinal uptake transporter PepT1 and the renal reabsorption transporter PepT2 [6–10]. Notably, these proteins do not only transport dietary peptides, but also a variety of peptidomimetic drugs and amino acid-conjugated pro-drugs, and are therefore not only physiologically important, but also of great pharmacological relevance [11,12].

PepTs are believed to function by an alternate access mechanism involving gated transitions between inward open, occluded and outward open conformational states [13], which is essentially similar to that of any other MFS transporter [14,15]. Due to the inherent difficulty in crystallization and structure determination of integral membrane proteins, the first PepT crystal structure, which represents PepT_So_ from *Shewanella oneidensis*, did not appear before 2011 [16]. However, in correlation with a general upsurge in MFS structure determinations [14,15], several additional structures soon followed [17–26]. Currently, structures are known for a total of six different bacterial PepTs, which were curiously all captured in inward facing conformations: PepT_So_ and PepT_So2_ from *Shewanella oneidensis*, PepT_St_ from *Streptococcus thermophilus*, GkPOT from *Geobacillus kaustophilus*, YbgH from *Escherichia coli*, and YePEPT from *Yersinia enterocolitica* [17–26]. Although much still remains to be understood, these structures have aided greatly in understanding substrate recognition and key aspects of the transport mechanism.

We set out to determine a structure of PepT_St_ before any other PepT structures were known. However, while our work was ongoing, several PepT structures were presented [17–26] including structures of inward facing PepT_St_ crystallized in space groups P2_1_2_1_2_1_ and C222_1_ [17,21,25,26]. The structure presented here is likewise inward facing, but belongs instead to space group P3_1_21. We describe in detail how this structure was obtained, in order to provide a case report that may be of use to others who wish to pursue structure determinations of MFS transporters. In addition, we compare all three crystal forms with the aim of furthering our understanding of the structural plasticity of PepT_St_ and its role in enabling the conformational cycle of the protein.

## Materials and methods

### Materials

The detergents used for purification were from Anatrace (Maumee, OH, USA), terrific broth (TB) was from Formedium (Norfolk, UK), kanamycin was from Duchefa Biochemie (Haarlem, NL), isopropyl β-D-1-thiogalactopyranoside (IPTG) was from Saveen Werner (Limhamn, S), and crystallization reagents were from Qiagen (Germantown, MD, USA). All other chemicals were of analytical grade and obtained from Sigma-Aldrich (St. Louis, MO, USA), unless otherwise stated.

### Expression and purification

**Fig 1:**
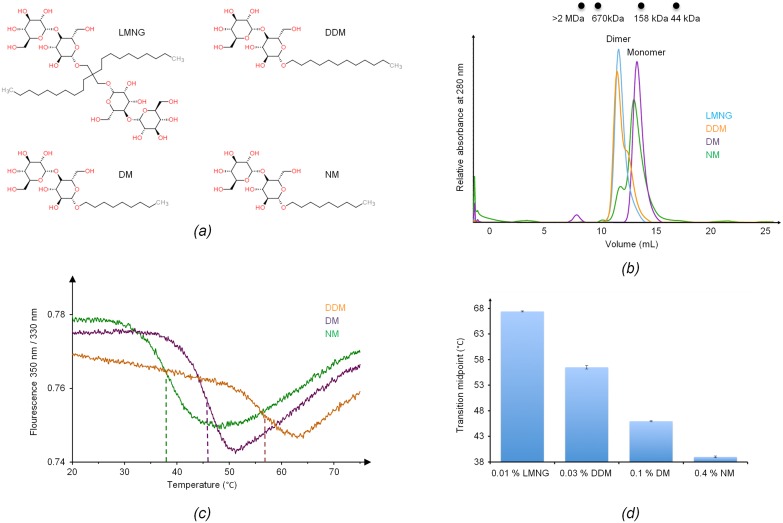
Detergent screening. *(a)* Chemical structures of the four different detergents used for purification: LMNG (Lauryl Maltose Neopentyl Glycol), DDM (n-Dodecyl-β-D-Maltopyranoside), DM (n-Decyl-β-D-Maltopyranoside), and NM (n-Nonyl-β-D-Maltopyranoside). *(b)* Size exclusion chromatography (SEC) profiles for PepT_St_ purified in LMNG (blue), DDM (orange), DM (purple), and NM (green). *(c)* Differential scanning fluorimetry results. Thermal stability curves of the protein in the presence of the detergents NM, DM and DDM are shown (same color code as in panel *b*). The fluorescence ratio (F_350nm_/ F_330nm_) is represented as a function of temperature. Dashed lines indicate the melting temperatures (T_m_). *(d)* Comparison of the T_m_ values for PepT_St_ purified in different detergents. The error bars were calculated from three independent measurements.

We have previously reported the identification of PepT_St_ as a suitable target for structural studies using a pipeline approach (referred to as Ce27 in that study) [27]. Here, we used the same C-terminally hexahistidine-tagged wild-type PepT_St_ construct, and furthermore prepared two mutant variants, L226M and F338M, using standard Quikchange protocols. Protein expression in *Escherichia coli* C41, membrane solubilization and purification using IMAC and size exclusion chromatography (SEC), was carried out as described [27], except that a partially different set of detergents were used. It may be noted that the protein was eluted from the IMAC column by passing TEV protease over the beads, as the usage of high imidazole concentrations for the elution step were found to strongly destabilize the protein (Löw et al., 2013). The detergents used were four different alkyl maltosides: n-dodecyl-β-D-maltopyranoside (DDM), n-decyl-β-D-maltopyranoside (DM), n-nonyl-β-D-maltopyranoside (NM), and the recently developed lauryl maltose neopentyl glycol (LMNG) [28] (Fig 1a). For membrane solubilization, the detergent concentration was 1%. For purification, we used 0.03% of DDM, 0.2% of DM, 0.4% of NM, or 0.01% of LMNG. For each purification campaign, the same detergent was used from solubilization to the final SEC step, except for NM. For this detergent, PepT_St_ was thus purified in DM up till the IMAC step, after which the detergent was exchanged to NM in the SEC step. For SeMet labeling, the protein was expressed in minimal medium supplemented with SeMet according to established protocols [29].

### Protein stability measurements

The stability of the different purified protein preparations was followed using a nanoDSF differential scanning fluorimeter (NanoTemper Technologies, GmbH). Here the intrinsic fluorescence at 330 and 350 nm after excitation at 280 nm is used to monitor the fluorescence change upon heat unfolding. Up to 48 samples can be measured in parallel without the addition of a dye. Typically, 10 μL of protein solution at a concentration of 0.5 mg/mL was loaded in a capillary (in triplicates), and the unfolding was then measured at a heating rate of 1°C/min. The first derivative of the unfolding curves was used to determine the transition midpoint. As control experiment, the concentration dependence of the transition midpoint was determined in the range of 0.2–5 mg/mL for each protein batch. Resulting transition midpoints were within 1°C.

### Crystallization procedures

Crystallization trials were carried out by vapor diffusion in 96-well sitting-drop plates at both 277 K and 293 K. The volume of crystallant added to the reservoir was 50 μL, while the drops had a total volume of 300 nL and were composed of the PepT_St_ sample in a concentration of 5–10 mg/mL, and the crystallant in ratios of 1:2, 1:1 or 2:1. Initial crystal hits were readily obtained regardless of whether the protein had been purified in DDM, DM or NM, but the best diffraction was attained when using NM, a temperature of 277 K, and a crystallant containing small PEGs as well as a buffer at near neutral to slightly alkaline pH (Table 1). Shifting to a 24-well format and scaling up the drop size was not found beneficial, which is an experience that we have also had with other integral membrane proteins [19,30,31]. For optimization, a wide range of conventional additives e.g. divalent cations were tested, along with numerous secondary detergents. Here, Fos-Choline-10 was found to improve diffraction significantly, and was therefore included in the crystallant (Table 1). We also tested the addition of peptides and other putative substrates. However, none of these improved the diffraction, and none could be identified in the electron density maps. This also includes the penicillin G compound that was present in the protein sample, which gave rise to the crystal used for refinement (Table 1). The crystals were typically flash frozen in liquid nitrogen without prior soaking in cryobuffer.

**Table 1:** Crystallization condition.

Method	Sitting-drop vapor diffusion
Temperature (K)	277
Protein concentration	8.5 mg/mL
Buffer composition of protein solution	20 mM HEPES pH 7.5, 150 mM NaCl, 5% glycerol, 0.4% NM, 0.75 mM TCEP, 10 mM Penicillin G
Composition of reservoir solution	0.05 M HEPES pH 8.0, 30% PEG550 MME, 1.2% Fos-Choline 10

### Data processing and experimental phasing

Four data sets were used for structure determination: a native one used for refinement, and another native as well as two derivatives used for MIRAS phasing (Table 2). All data sets were processed using the XDS package [32]. As the diffraction displayed severe anisotropy, the native data sets were furthermore subjected to anisotropic scaling and truncation using the Diffraction Anisotropy Server [33] (Table 2). Initial phasing was carried out using a trimmed version of the PepT_So_ structure (PDB: 2XUT) as a molecular replacement search model in Phaser [34] from the PHENIX suite [35], but the map was not of high quality, and we therefore decided to pursue experimental phases. The derivatives used for phasing were a SeMet labeled F338M crystal and a wild-type crystal soaked with KAu(CN)_2_. Soaking was carried out by adding 1.5 μL of a solution containing 90% of the original crystallant and 1 mM KAu(CN)_2_ to a crystallization drop with a well-formed crystal, which was then removed and flash frozen in liquid nitrogen after an incubation time of nine minutes. Scaling, site finding and initial MIRAS phasing was carried out with AutoSHARP [36], while final sub-structure refinement, phasing and density modification was carried out with SHARP [37] (Table 3). We used an initial resolution range of 48.94–4.10 Å, corresponding to the full range of the SeMet data set, and also close to that of the KAu(CN)_2_ data set. This was however extended to the full range of the native data set, 48.94–3.60 Å, during density modification. FOM was 0.401 for acentric reflections and 0.258 for centric reflections before density modification, and respectively 0.281 and 0.204, after.

**Table 2:** Data collection and processing. The Values for the outer shell are given in parentheses. The native data sets were subjected to anisotropic scaling and truncation (see main text for details). Without truncation 〈*I*/σ(*I*) of the native data set used for refinement falls below 2.0 between a maximum resolution of 3.80 and 3.75 Å at an overall completeness of over 99%.

Crystal	Native 1 (for refinement)	Native 2 (for phasing)	Derivative 1 (L338M SeMet)	Derivative 2 (KAu(CN)_2_)
Diffraction source	Diamond I04	Diamond I24	Diamond I24	Diamond I02
Wavelength (Å)	0.9795	1.0000	0.9788	0.8920
Temperature (K)	100	100	100	100
Space group	P3_1_21	P3_1_21	P3_1_21	P3_1_21
*a*, *b*, *c* (Å)	80.19, 80.19, 293.69	79.51, 79.51, 293.64	81.37, 81.37, 294.57	81.10, 81.10, 293.25
α, β, γ (°)	90, 90, 120	90, 90, 120	90, 90, 120	90, 90, 120
Resolution range (Å)	44.85–3.40 (3.49–3.40)	44.68–3.60 (3.69–3.60)	45.20–4.10 (4.21–4.10)	48.88–4.20 (4.31–4.20)
Total no. of reflections	83693 (601)	148684 (6829)	179388 (13126)	185686 (13620)
Completeness (%)	74.6 (5.8)	97.1 (65.1)	99.6 (99.4)	99.2 (98.6)
Redundancy	5.3 (0.5)	11.2 (7.2)	10.5 (10.5)	11.8 (11.4)
〈*I*/σ(*I*)〉	13.67 (3.13)	19.40 (2.49)	17.75 (2.95)	12.31 (3.35)
*R*_meas_	8.9 (67.9)	7.2 (106.5)	7.1 (124.9)	8.5 (104.4)
Wilson *B-*factor (Å^2^)	96.4	129.16	156.5	174.2

**Table 3:** MIRAS phasing statistics.

Derivative	SeMet		KAu(CN)_2_	
Resolution (Å)	48.94–4.10		48.94–4.10	
Number of sites found	20		4	
Type of reflections	Acentric	Centric	Acentric	Centric
Phasing power (isomorphous)	0.801	0.700	1.312	1.859
Phasing power (anomalous)	1.784	0.000	0.325	0.000
*R-*Cullis (isomorphous)	0.403	0.478	0.200	0.159
*R-*Cullis (anomalous)	0.624	0.000	0.987	0.000

### Refinement and structural analysis

Model building and rebuilding was carried out in Coot [38]. We initially relied only on the experimental map, but later also used the P2_1_2_1_2_1_ and C222_1_ structures for guidance as they became available (Solcan *et al*., 2012; Lyons *et al*., 2014). Refinement was carried out with PHENIX refine [35] using one translation libration screw (TLS) group (Table 4). In order to ensure good geometry of the model, both secondary structure and reference restraints were used. This was found to greatly improve the Ramachandran plot and to reduce the number of rotamer outliers. For the last rounds of refinement, the X-ray/stereochemistry and X-ray/ADP weights were furthermore optimized, which helped improve RMS bonds and angles, as well as the Molprobity clash score [39]. The final clash score was 6.8 and the overall Molprobity score was 1.4 (both are in the 100^th^ percentile of structures with similar resolution). Structural alignments were made using the DALI server (http://ekhidna.biocenter.helsinki.fi/dali_server/start) [40], and structure figures were generated using PyMol [41].

**Table 4:** Refinement statistics.

Resolution range (Å)	44.85–3.40
No. of reflections, working set	11292
No. of reflections, test set	589
Final *R*_cryst_	0.265
Final *R*_free_	0.286
No. of non-H atoms	
Protein	3283
Other	0
R.m.s. deviations	
Bonds (Å)	0.005
Angles (°)	1.188
Average *B* factors (Å^2^)	127.1
Ramachandran plot	
Most favoured (%)	99.3
Allowed (%)	0.7

## Results and discussion

### Characterization of the oligomeric state and thermal stability in different detergents

In a previous high-throughput screening study, we found that maltoside detergents are suitable for purification of PepT_St_ [27]. Here we screened four maltosides with aliphatic chains of different lengths: DDM, DM and NM, which have chain-lengths of twelve, ten and nine, respectively, as well as LMNG, which chemically represents a fusion of two DDM molecules (Fig 1a). These detergents all supported good purification yields and did not cause significant aggregation, as evaluated by SEC (Fig 1b). Using blue native PAGE and chemical cross-linking, we have previously shown that PepT_St_ forms dimers in DDM [19]. The SEC results provided here show that LMNG also supports the dimeric form, while the shorter-chain detergents DM and NM disrupt it (Fig 1b). This is not a unique case. The higher oligomeric forms of rhodopsin were thus likewise found to be disrupted when substituting long-chain maltoside detergents with shorter-chain variants [42]. We next measured the thermal stability of PepT_St_, using intrinsic fluorescence measurements. Protein stability generally decreases upon shortening of the alkyl chain of the detergent used for purification, and this was also found to be the case for PepT_St_. Indeed, the effect was quite dramatic, with transition midpoints for thermal unfolding spanning a temperature range of 39°C in NM to almost 69°C in LMNG (Fig 1c and 1d). Finally, we also analyzed the thermal stabilities of SeMet labeled variants of the wild-type protein and two mutants, L226M and F338M, and found them to be very similar to unlabeled wild-type PepT_St_ (data not shown).

### Obtaining the P3_1_21 crystal form

Crystallization trials were carried out for protein purified in DDM, DM and NM, and yielded crystal hits in all three cases. We obtained the best initial diffraction when using NM, though others have obtained similar resolution for a different crystal form, P2_1_2_1_2_1_, which was grown using DDM [17]. As mentioned above, NM is highly destabilizing compared to the longer-chain maltoside detergents, and it may therefore seem counterintuitive that it yields good crystals. However, although the stabilizing effect of detergents generally decreases with shorter chain-length, the micelle size also decreases, which can potentially support tighter crystal packing in some directions [43]. Indeed, a recent survey of the alpha-helical membrane protein structures in the Protein Data Bank (PDB) confirms that the alkyl chain-length of the detergent used for crystallization is on average inversely correlated with diffraction potential [44]. It is therefore generally advisable to test short-chain detergents for crystallization, even when they are highly destabilizing compared to longer-chain variants, provided that they do not cause significant aggregation during purification or concentration of the purified sample. The use of secondary detergents for purification or as crystallization additives can have a crucial effect on diffraction quality, as is for example well illustrated by the case of the structure determination of cc3b cytochrome c oxidase [45], and their use is therefore becoming increasingly common [44]. For this reason we tested numerous detergent additives, and found that Fos-Choline-10 significantly improved the diffraction potential.

### Structure determination

Native crystals diffracted to 3.4 Å maximum resolution, but in a severely anisotropic fashion. The data were therefore truncated and scaled anisotropically. As cut-off values for ellipsoidal truncation, we used the values suggested by the Diffraction Anisotropy Server [33], which were 3.9 Å in the *a*-direction, 3.7 Å in the *b*-direction and 3.4 Å in the best diffracting *c*-direction of the crystal. The resolution is similar to what was obtained for the P2_1_2_1_2_1_ form (3.3 Å anisoptropic) [17], while the C222_1_ form diffracted considerably better (up to 2.3 Å isotropic) [21,25]. At the time the native crystals were optimized, the first structure of PepT_So_ had recently been published, and was therefore tested as search model for molecular replacement. A solution could indeed be found, which was essentially correct, as evidenced by anomalous difference Fourier maps calculated using preliminary SeMet data. However, as the map was of poor quality, and as model bias can be severe at the resolution we were able to attain, we decided to move on to experimental phasing using SeMet and heavy metal derivatives. In order to ensure accurate sequence assignment, we originally planned to generate a series of methionine mutants, and started out by introducing L226M and F338M point mutations in regions that were poorly defined in preliminary maps. However, when the P2_1_2_1_2_1_ and C222_1_ PepT_St_ structures were published, sequence assignment became a trivial matter and the strategy was therefore abandoned. Nonetheless, the best SeMet data set was obtained using the F338M mutant (Table 2). For heavy metal derivatization, numerous Hg, Au, Pt, Pb and lanthanide salts were tested in both co-crystallization and soaking experiments. However, we were only successful when soaking with KAu(CN)_2_, which, incidentally, is one of the most commonly used heavy metal salts for phasing of both soluble and membrane imbedded proteins [46].

**Fig 2:**
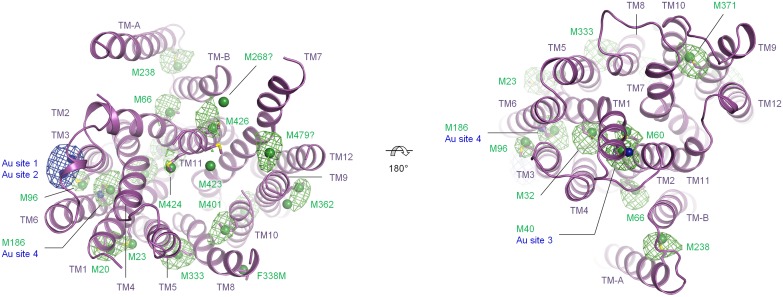
Site finding. The structure is magenta and the methionine side chains are shown in sticks and labeled, green and blue spheres designate AutoSHARP SeMet and Au sites, respectively, and the similarly colored wire meshes designate the model phased SeMet and Au anomalous difference Fourier maps contoured at 3 σ. Two views are shown: cytoplasmic (left) and periplasmic (right).

**Fig 3:**
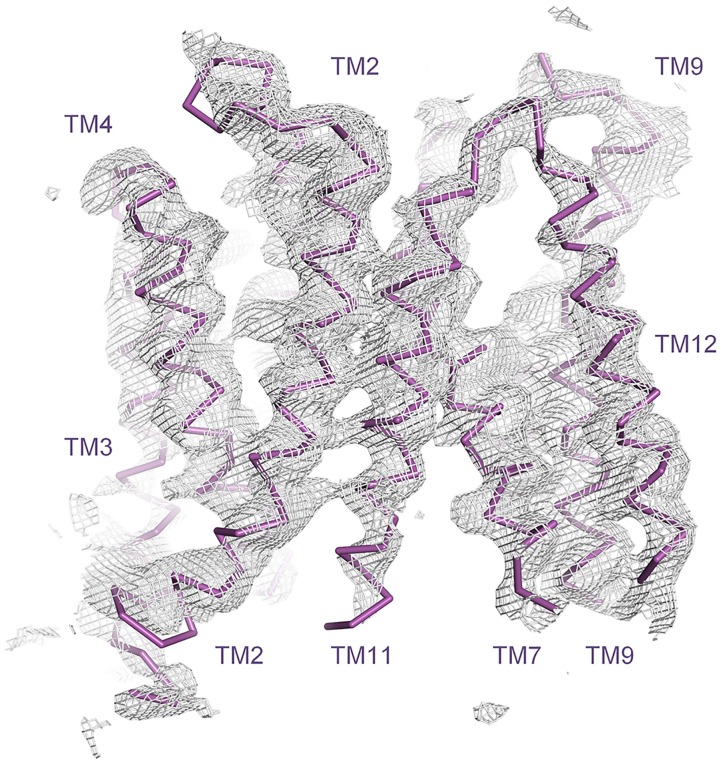
Experimental electron density map. The structure is shown in magenta, and the density modified MIRAS phased electron density map is shown at 1 σ as a grey wire mesh.

AutoSHARP [36] was found to exhibit impressive sensitivity with respect to site finding. SeMet sites were thus identified for all methionines modeled in the structure, M20, M23, M32, M40, M60, M66, M96, M186, M238, M333, F338M, M362 (two sites), M371, M401, M423 and M424, as well as for what appears to be the unmodeled methionines, M268 and M479 (Fig 2). AutoSHARP uses log-likelihood gradient maps in the site finding procedure, which are more sensitive than anomalous difference Fourier maps. Indeed, for some of the identified sites, no difference Fourier peak could be detected at a threshold of 3 σ in maps calculated using model phases—even when these model phases were derived from the final refined structure (Fig 2). We suggest bearing this in mind for other projects where SeMet is to be used for chain tracing. As for the Au sites, two of them share a substantial anomalous difference peak in a hydrophobic pocket located between the cytoplasmic ends of transmembrane helices 3 and 6 (TM3 and TM6), and two more are found near M40 and M186, respectively, where, in contrast, very little difference density is observed (Fig 2). The SeMet and Au data sets were used together with an appropriate native data set to generate MIRAS phases in SHARP (Table 3) [37]. The transmembrane helices were generally well defined in the experimental map (Fig 3). There are in total fourteen of them, which are arranged in the same overall pattern as described previously [17]. Briefly, TM1–TM6 forms an N-terminal MFS domain, termed the N-domain, TM7–TM12 forms a similar C-terminal MFS domain, termed the C-domain, and the last two helices, TM-A and TM-B, are found in the linker between the two domains, where they adopt a V-shaped arrangement (Figs 2 and 3). As mentioned above, an anisotropically corrected data set was used for refinement. This was found to perform well (Table 4), in spite of exhibiting low completeness as measured within circular resolution shells (Table 2).

### Crystal packing

**Fig 4:**
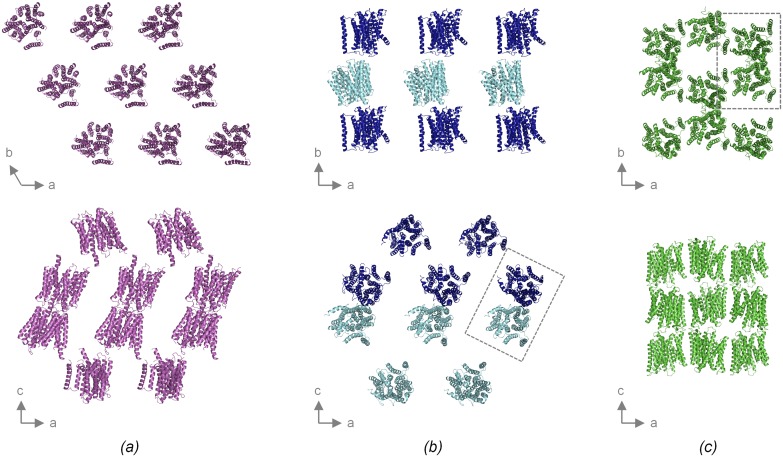
Comparisons of crystal packing. *(a)* Crystal packing in the P3_1_21 form. Top: slice of the crystal taken parallel to the *(a-b)*-plane and encompassing one layer of molecules. The PepT_St_ molecules are colored magenta. Bottom: slice of the crystal taken parallel to the *(b-c)*-plane. *(b)* Crystal packing in the P2_1_2_1_2_1_ form (PDB: 4APS). Depicted as in panel *a*, except that the two molecules in the asymmetric unit are colored light and dark blue, and that the dimer formed by these are boxed. *(c)* Crystal packing in the C222_1_ form (PDB: 4D2C). Depicted as in panel *a*, except that the molecules are colored green, and that a dimer with the same arrangement as seen in the P2_1_2_1_2_1_ form is similarly boxed, though this dimer is generated by crystal symmetry rather than being non-crystallographic. Crystal packing interfaces are shown in S1 Fig.

The packing modes in the three crystal forms are very different from each other (Fig 4). However, a common feature of the P3_1_21 and P2_1_2_1_2_1_ forms is that they both exhibit ordered end-to-end packing through periplasmic-periplasmic and cytoplasmic-cytoplasmic interactions, while ordered lateral contacts are entirely missing (Fig 4a and 4b). Loose lateral packing is a typical feature of membrane protein crystals grown using traditional *‘in surfo’* vapor diffusion methods and relates to the sometimes prohibitive effect of the detergent belt on forming tight contacts [47]. A total lack of ordered lateral contacts is rather extreme, but not unheard of. Similar cases have thus been reported for other membrane proteins, such as the MFS transporter XylE and the porin OmpF [48,49]. Notably, a lack of ordered lateral contacts typically manifests itself during data collection as severe diffraction anisotropy [48,50]. As mentioned above, such severe anisotropy was indeed also observed for the P3_1_21 and P2_1_2_1_2_1_ forms of PepT_St_. Moreover, in both cases the directions exhibiting the most dramatic fallout of reflection intensity, do indeed correlate with the directions in the crystals where ordered packing interactions are missing (data not shown). The C222_1_ form stands out by encompassing tight lateral interactions, causing the formation of continuous layers, which pack against each other in an overlapping fashion through head-to-tail interactions between periplasmic and cytoplasmic parts (Fig 4c). This results in a quite compact packing pattern, which no doubt underlies the much better resolution and more isotropic diffraction of this crystal form. Tight packing is possible here, since crystallization was performed *in meso*, causing the detergent belt to be replaced with lipids, which better mimic the plasma membrane, and supports rather than inhibits packing interactions between transmembrane parts [51].

The packing interfaces in the three crystal forms are almost invariantly different and are also formed by somewhat different subsets of structural elements (S1 Fig). The P2_1_2_1_2_1_ and C222_1_ forms do however share a single interface, though it is non-crystallographic rather than crystallographic in the former case. This interface is mainly formed by TM1, TM5 and TM6 (S1 Fig), and generates a specific side-by-side antiparallel dimer that is not seen in the P3_1_21 form (Fig 4). As mentioned previously, PepT_St_ forms dimers in DDM, which was used for purification in the cases of the C222_1_ and P2_1_2_1_2_1_ forms, but not in NM, which was used for the P3_1_21 form (Fig 1b). It thus seems likely that the dimer seen in the crystal is the same as observed in solution. Some PepTs probably function as homooligomers [19], but so far, no attempts have been made to characterize the oligomeric state of PepT_St_ in the membrane, and it is therefore unknown if a monomeric or oligomeric form is preferred *in vivo*. Yet, we find it rather unlikely that this particular dimer is physiologically relevant. An antiparallel arrangement with one protomer being flipped in the membrane to expose the cytoplasmic face to the extracellular side and the extracellular face to the cytoplasmic side does thus not seem likely to be functionally productive. Moreover, we are not aware of any functional antiparallel MFS dimers having ever been reported. However, regardless of the lack of an obvious biological relevance, the formation of the dimer may facilitate crystal nucleation, as homo oligomers tend to crystallize more readily than monomers [52], and would also in part dictate the overall packing pattern. In fact, it is likely that the difference in crystal packing between the three crystal forms are to a significant extent governed by the differences in oligomerization combined with the above mentioned differences in crystallization methods and their associated differential effects on packing between the transmembrane regions.

### Structural variation and flexibility

**Fig 5:**
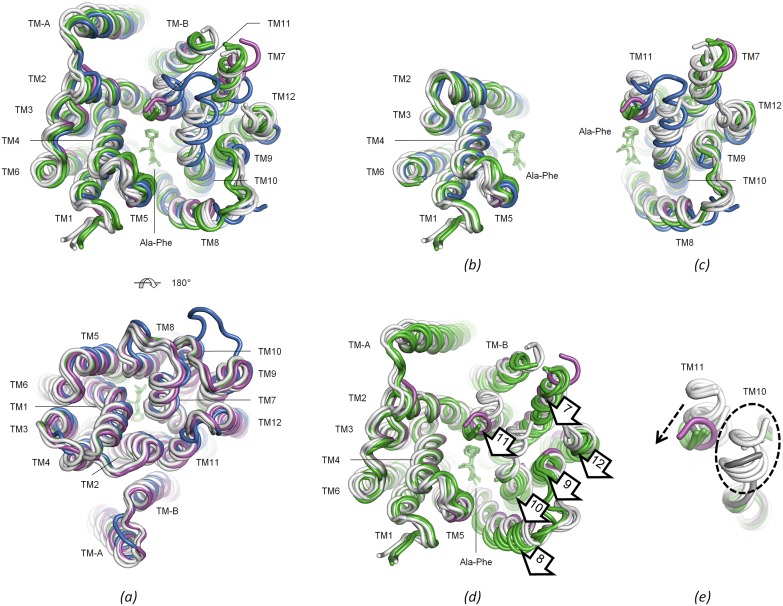
Structural comparisons. *(a)* All previously determined PepT_St_ structures superimposed on the P3_1_21 structure (see also Table 5). The P3_1_21 structure is magenta, the P2_1_2_1_2_1_ structure is blue the C222_1_ structures with dipeptides are green (dipeptides are shown in sticks), and the C222_1_ structures without dipeptides are white. Two views are shown—cytoplasmic (top) and periplasmic (bottom). *(b)* Superimposition of the N-domains of the same structures used in panel *a*. *(c)* Superimposition of the C-domains of the same structures used in panel *a*. *(d)* Same as in panel *b*, except that the C-domains are shown alongside the superimposed N-domains and that the P2_1_2_1_2_1_ structure was omitted. A rotation of the C-domain relative to the N-domain can be recognized for the C222_1_ structures with dipeptides (rotation marked with arrows). (e) Same as in panel *c*, except that only TM10 and TM11 are shown, that the C222_1_ structure determined at room temperature is grey, and that the P2_1_2_1_2_1_ structure was omitted. The C222_1_ structures with dipeptides, the P3_1_21 structure and to some extent the C222_1_ structure determined at room temperature, show bending of TM11 (indicated with a dashed arrow) and increased disorder in TM10 (indicated with a dashed oval) relative to the other C222_1_ structures determined at cryogenic temperatures.

The P2_1_2_1_2_1_ structure can be superimposed on the P3_1_21 structure with an RMSD value of 1.4 Å over 409 out of 425 C-alpha atoms (Table 5). For the individual domains, the values are 1.0 Å over 190 out of 190 C-alpha atoms for the N-domain, and 1.7 Å over 160 out of 174 C-alpha atoms for the C-domain (Table 5). The C-domain thus varies more than the N-domain between these two structures. The most obvious structural differences are in loop regions. For example, the loop between TM9 and TM10 differs markedly between the two structures, and the lobe encompassing the cytoplasmic tips of TM10 and TM11 as well as the connector loop between them is much better ordered in the P2_1_2_1_2_1_ structure than in the P3_1_21 structure (Fig 5a–5c). However, there are also some smaller differences in position or bending of TM7, TM9 and TM12 in the C-domain (Fig 5c).

**Table 5:** Overview of previously determined PepT_St_ structures and their structural similarity to the P3_1_21 structure reported in this study.

Crystal form	PDB	Ligand modeled	Data collection	Superimposition on the P3_1_21 structure					
				Full-length		N-domain		C-domain	
				RMSD (Å)	No. C-alphas	RMSD (Å)	No. C-alpha	RMSD (Å)	No. C-alphas
P2_1_2_1_2_1_	4APS	None	Loop, 100 K	1.4	409	1.0	190	1.7	160
C222_1_	4D2C	Dipeptide	Loop, 100 K	0.7	413	0.7	190	0.5	162
C222_1_	5D58	Dipeptide	Plate, 100 K	0.9	423	0.7	190	0.7	172
C222_1_	5D59	Dipeptide	Loop, 100 K	0.9	423	0.7	190	0.6	172
C222_1_	4D2D	Tripeptide	Loop, 100 K	0.9	424	0.6	190	1.1	173
C222_1_	4D2B	None	Loop, 100 K	1.0	420	0.6	190	1.4	170
C222_1_	4XNI	None	Plate, 293 K	0.8	417	0.5	190	0.7	166
C222_1_	4XNJ	None	Loop, 100 K	0.9	425	0.6	190	1.6	154

Seven C222_1_ structures have been published [21,25,26], which differ in which substrate were used for crystallization, in how the data were collected, and in which temperature was used during data collection (Table 5). They are all structurally rather similar, though as remarked by others [21], the structures with a dipeptide in the binding site differ somewhat from the other structures determined at cryogenic temperatures by exhibiting a slightly different position of the C-domain relative to the N-domain (Fig 5d), and a marked bending of lobe TM10–TM11 towards the bound substrate as well as increased disorder at its apex, permitting fewer residues to be modeled (Fig 5e). The single structure determined at room temperature was obtained without a dipeptide, but interestingly also displays some bending of TM11 (Fig 5e), as has likewise been remarked by others [25]. The P3_1_21 structure is more similar to any of the C222_1_ structures than it is to the P2_1_2_1_2_1_ structure. The full-length proteins can thus be superimposed with RMSD values ranging from 0.7–1.0 Å over 413–425 C-alpha atoms, the N-domains with values of 0.5–0.7 Å over 190 C-alpha atoms, and the C-domains with values of 0.5–1.6 Å over 154–173 C-alpha atoms (Table 5). The structural overlays reveal differences in loop TM3–TM4, loop TM4–TM5 and loop TM9–TM10, as well as in the bending of the cytoplasmic part of TM7 (Fig 5a–5b). Most of these differences could however be caused by packing effects, as all these structural elements are also implicated in crystal packing in one or both of the crystal forms (S1 Fig). Thus, although crystal contacts are not generally a major source of imprecision in crystal structures [53,54], smaller perturbations across such interfaces are not unlikely to occur [55,56]. Of more interest, the P3_1_21 structure exhibits a marked bending of TM11, which is highly reminiscent of the bending observed in the C222_1_ structures with dipeptides (Fig 5e). Furthermore, the tip of TM10 is also bent in much the same way as in these structures, and is about equally disordered (Fig 5e). This is also reflected in the RMSD values (Table 5)–the C-domain of the P3_1_21 structure thus superimposes on the C222_1_ structures with dipeptides with RMSD values of 0.5–0.7 Å over 162–172 C-alpha atoms, while it superimposes on the other C222_1_ cryogenic structures with noticeably higher values of 1.1–1.6 Å over 154–173 C-alphas. On the other hand, the domain rotation observed in the C222_1_ structures with dipeptide does not apply to the P3_1_21 structure (Fig 5d). The P3_1_21 structure thus adopts what can be viewed as a hybrid conformation, which shares the bending of lobe TM10–TM11 with the C222_1_ structures with dipeptides, but the orientations of the domains relative to each other with the C222_1_ cryo structures without dipeptides. Notably, the C222_1_ structure determined at room temperature adopts a somewhat similar hybrid conformation, though lobe TM10–TM11 is less bent here (Fig 5e). Based on these observations, we infer that lobe TM10–TM11 can undergo bending independently of domain rotation, and that it samples a range of differently bent positions, rather than simply snap back and forth between discrete bent and unbent positions.

**Fig 6:**
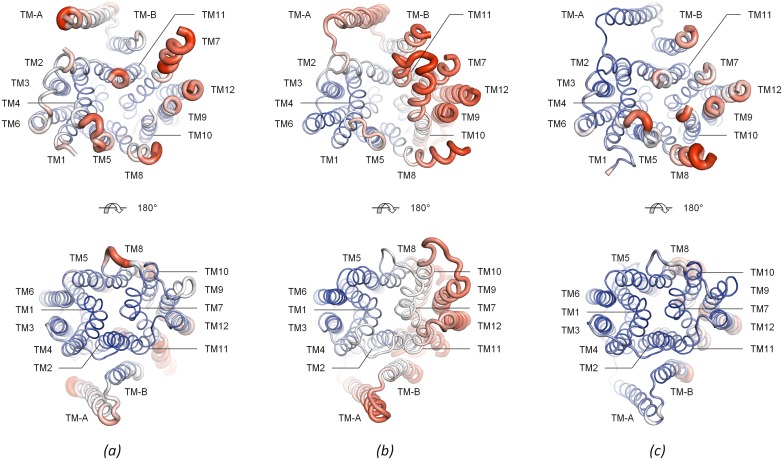
*B-*factor analysis of inward facing PepT_St_. *(a)* The P3_1_21 structure. The structure is shown in a putty tube representation where the diameter of the tube is correlated with the magnitude of the C-alpha *B-*factors. The structure is furthermore also colored by C-alpha *B-*factors: colors are ramped from blue over white to red, with blue designating low values and red designating high values. Two views are shown: cytoplasmic (top) and periplasmic (bottom). *(b)* The P2_1_2_1_2_1_ structure (PDB: 4APS) depicted as in panel *a*. *(c)* The C222_1_ structure (PDB: 4D2C) depicted as in panel *a*. A similar *B-*factor analysis on PepTs from other organisms is provided in S2 Fig. Higher *B-*factors are an indication of higher flexibility, but it should be noted that the regions with highest flexibility are those that could not be modeled at all, for example lobe TM10–TM11 in the C222_1_ and P3_1_21 structures, and the cytoplasmic tip of TM7 in the P2_1_2_1_2_1_ and C222_1_ structures.

Analyzing how the crystallographic *B-*factors vary along the sequence is a commonly used procedure for identifying putative flexible regions. However, one should bear in mind that *B-*factors are sensitive to modeling errors [57], and can be heavily influenced by crystal contacts [58,59]. It is therefore desirable to include as many crystal forms as possible in such an analysis. Based on *B-*factor analysis, structural comparisons, and molecular dynamics studies, it has previously been inferred that the helices in the C-domain of PepT_St_ are more flexible than those in the N-domain [17,21,23]. Here we show that an expanded *B-*factor analysis, which includes the monomeric P3_1_21 crystal form, further supports this assertion (Fig 6). Specifically, the cytoplasmic side of the C-domain exhibits particularly high *B-*factors in all three crystal forms of inward facing PepT_St_ (Fig 6). This is of course well in line with the results from the structural comparisons of the different crystal forms, which as mentioned above identified the C-domain as the source of most of their structural differences. It should however be mentioned that the *B-*factor distribution is considerably less consistent if also including the other PepTs in the analysis (S2 Fig). Zones with particularly high *B-*factors are for example basically absent in GkPOT, YbgH and YePEPT, if exempting a couple of connectors (S2 Fig). At present, it is therefore unclear to which extent flexible regions may be conserved across the PepT family.

### Role of lobe TM10–TM11 in forming the inward facing occluded state

Two different occluded conformations have been identified for MFS transporters—outward facing occluded and inward facing occluded [15]. These states are both formed by bending of one or more pore lining helices towards the binding site, thus forming a constriction (typically referred to as a ‘gate’) that prevents substrate from entering or leaving [15]. Specifically, the outward facing occluded state is formed by bending of one or more of TM1, TM2, TM7 and TM8 on the extracellular side, and the inward facing occluded state by bending of one or more of TM4, TM5, TM10 and TM11 on the cytoplasmic side [15]. The bending of lobe TM10–TM11 towards the binding site in the P3_1_21 and the C222_1_ structures with bound dipeptides thus indicates that these structures are either inward facing occluded states or intermediates between occluded and fully inward open, though it cannot presently be ascertained which of these options applies due to much the lobe being disordered. Notably, a central role of lobe TM10–TM11 in occluding the binding site from the cytoplasmic side is not a novel proposition, but has also been suggested previously based on structural comparisons between PepT_St_ and PepT_So_ (characterized as inward facing occluded) [17], comparisons of the individual C222_1_ PepT_St_ structures [21], and through molecular dynamics studies carried out on PepT_St_ and PepT_So_ [23]. Bending of lobe TM10–TM11 may however not act alone in forming the fully occluded inward facing state. As mentioned above, a slight domain rotation can thus be observed in the C222_1_ structures with dipeptides, which is likely also significant. Furthermore, structural changes in lobe TM4–TM5 and the TM-A and TM-B helix pair could potentially also be involved, as discussed below.

### Functional roles of the A-motif and the TM-A and TM-B helix pair

**Fig 7:**
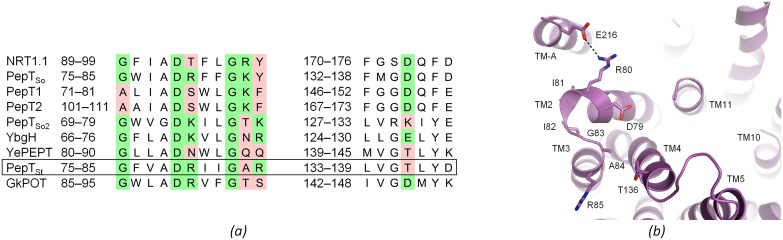
The A-motif. *(a)* Sequence alignment. Two fragments are shown of a sequence alignment that includes PepT_St_ (boxed), the other PepTs for which structures are known, the related nitrate transporter NRT1.1 from *Arabidopsis thaliana*, for which structures are also known, and human PepT1 and PepT2. The left alignment fragment covers the A-motif in loop TM2–TM3, and the right fragment covers the region around the co-conserved acidic residue in TM4. Residues that match the consensus sequence G-X-X-X-(D/E)-(R/K)-X-G-[X]-(R/K)-(R/K) are highlighted in green, while those that do not are highlighted in pink. *(b)* Structure of the A-motif and the surrounding region, as modeled in the P3_1_21 structure of PepT_St_. Residues constituting the A-motif are labeled, and a salt bridge between R80 in the A-motif and E216 in TM-A is indicated with dashes.

The A-motif represents the most well conserved motif in MFS transporters [60,61]. It has the consensus sequence G-X-X-X-(D/E)-(R/K)-X-G-[X]-(R/K)-(R/K) and typically spans loop TM2–TM3 and/or loop TM8–TM9 on the cytoplasmic side [60,61]. It functions in controlling the inward-outward transition, with the acidic residue capping a helix on the opposite domain in the outward open state, while being disengaged in the inward open state [62]. Indeed, mutating the acidic residue in the peptide transporter YbgH resulted in reduced activity [22], echoing similar results obtained for other MFS transporters [62–64]. In both these states, the second basic residue furthermore interacts with a co-conserved acidic residue, which is found in TM4 (A-motif in loop TM2–TM3) or TM10 (A-motif in loop TM8–TM9) [62]. We recently found that a third state of the A-motif can be recognized in several inward facing occluded structures, in which this interaction is broken, thus allowing TM4 (A-motif in loop TM2–TM3) or TM10 (A-motif in loop TM8–TM9) to bend towards the pore and occlude the binding site [15]. In the PepTs, an A-motif is found in loop TM2–TM3 (Fig 7a). It differs however somewhat from the consensus sequence in having an extra residue inserted prior to the second conserved glycine [22], and by often missing one or more of the basic residues as well as the acidic residue in TM4 (Fig 7a). The second basic residue and the acidic residue in TM4 are for example both missing in PepT_St_ (Fig 7). The salt bridge normally found between these residues is thus absent here, which may suggest that regulated bending of TM4 does not take place in PepT_St_. However, it is possible that other interactions may substitute for this salt bridge, as for example seen in the nitrate transporter NarK [15,65]. Indeed, the second basic residue is also missing in GkPOT, and yet, molecular dynamics studies suggested that the inward facing occluded form of this protein is generated through bending of lobe TM4–TM5 [18]. It may be mentioned though that although an inward facing occluded MFS structures has been described where only lobe TM10–TM11 is bent, namely XylE [31], we are not aware of any where only lobe TM4–TM5 is bent [15]. Thus, although lobe TM4–TM5 is quite bent in the inward facing occluded structures of EmrD and PiPT, these structures also feature apparent bending of lobe TM10–TM11 [66,67]. We therefore speculate that if bending of lobe TM4–TM5 takes place in some or all PepTs, it probably happens in addition to rather than instead of bending of lobe TM10–TM11.

It has been suggested that the TM-A and TM-B helix pair characteristic of bacterial PepTs acts as a sensor of conformational changes in concert with the A-motif [22,62]. In this respect it is notable that these two structural elements are in close proximity to each other (Fig 7b). Indeed, the A-motif is generally in direct contact with loop TM6–TM-A, though the specific interactions formed are quite variable among the different PepTs (not shown). A salt bridge between the A-motif and TM-A could furthermore be identified in PepT_St_ (Fig 7b). However, although this interaction is also found in GkPOT, it is absent in all the other PepT structures, suggesting that it is either of little functional significance or that any function it may have in mediating communication between the A-motif and TM-A is replaceable. In conclusion, the transition of PepT_St_ from inward open to inward facing occluded involves bending of lobe TM10–TM11 and a slight domain rotation, as discussed above. However, bending of lobe TM4–TM5 may also play a role in at least some PepTs. Finally, the A-motif probably functions in regulating transitions between the inward and outward facing states, but may also have additional roles in regulating the conformational changes in PepTs.

## Conclusions and outlook

Here we have provided a report of the structure determination of PepT_St_ in space group P3_1_21, which may aid in guiding others aiming to determine structures of MFS transporters or other membrane proteins. Three different maltoside detergents were tested for crystallization. The best crystals obtained were grown using NM, which has the shortest chain-length, and is by far the most destabilizing of the three. This underlines that short-chain detergents should be tested for crystallization even if they are highly destabilizing compared to their longer-chain counterparts, as long as they do not cause significant aggregation. It should be mentioned though, that impressive results on PepT_St_ and other integral membrane proteins have been achieved using the increasingly popular *in meso* method [21,51]. For future projects we therefore suggest to use this technique in parallel to the traditional *in surfo* vapor diffusion method employed in this study.

The three different crystal forms now available for PepT_St_ exhibits very different packing patterns, which is probably in large part due to the fact that different methods were used for crystallization: *in surfo* for the P3_1_21 and P2_1_2_1_2_1_ forms, and *in meso*, which enables tighter packing of transmembrane parts, for the C222_1_ form, combined with the fact that the protein was purified in different detergents, favoring a monomeric form in the case of the P3_1_21 crystal form, but a likely unphysiological antiparallel dimer in the cases of the P2_1_2_1_2_1_ and C222_1_ forms. The P3_1_21 form is more similar to the C222_1_ structures than to the P2_1_2_1_2_1_ structure. It adopts an inward facing conformation that is either occluded or intermediary between inward open and occluded, sharing the bent position of lobe TM10-TM11 with the C222_1_ structures with peptides and the relative domain orientation with those without, thus indicating that bending of lobe TM10–TM11 can occur independently of domain rotation upon formation of the inward facing occluded state.

Much still remains to be understood about conformational changes in PepTs, which relates in part to the fact that all known PepT structures are inward facing. Obtaining structures of outward facing forms would thus clearly be highly valuable. Furthermore, spectroscopic methods such as single molecule Förster resonance energy transfer (FRET) or double electron-electron resonance (DEER) also hold great potential for further elucidating the conformational changes. Indeed, DEER spectroscopy applied to PepT_So_ and combined with *in silico* modeling and molecular dynamics studies has already helped in characterizing the outward open form of this protein [23]. It may be noted that PepT_St_ was found to be unsuitable for such a spectroscopic approach, as the dimeric nature of the purified protein would complicate data interpretation [23]. The protocol we have established here for purification of the monomeric form may thus prove useful for carrying out future spectroscopic studies and potentially other *in vitro* experiments on PepT_St_.

## Supporting information

S1 Fig Crystal packing interfaces. *(a)* The P3_1_21 structure. Coloration is by residue count with colors ramped from white (N-terminus) over wheat, golden and orange to red (C-terminus). Side chains within 4 Å of a symmetry related molecule are shown in sticks with transparent surfaces, and the structural elements in which they occur are labeled. Two side views are shown. The contact areas on the periplasmic side are much more extensive than on the cytoplasmic side. Specifically, the former involves loop TM1–TM2, loop TM5–TM6, loop TM7–TM8, loop TM9–TM10 and loop TM11–TM12, and the latter TM7 and loop TM4–TM5. *(b)* The P2_1_2_1_2_1_ structure (PDB: 4APS). Shown as in panel *a*, except that resides within 4 Å of the other molecule in the asymmetric unit are shown in light blue sticks with transparent surfaces. The contact surfaces encompass fewer residues here, and they are more evenly distributed between the periplasmic and cytoplasmic sides. Specifically, they are found in loop TM1–TM2 and loop TM11–TM12 on the former side and loop TM6–TM-A on the latter *(c)*. The C222_1_ form (PDB: 4D2C). Shown as in panel *a*. Two lateral interfaces are found. One is formed by the N-terminal tail, TM1, TM5, TM6, TM8 and loop TM7–TM8 packing against the equivalent elements of a symmetry-related mate to generate an antiparallel dimer, which is structurally equivalent to the non-crystallographic dimer seen in the P2_1_2_1_2_1_ form. These dimers are in turn linked into a continuous planar layers (Fig 4c, bottom) by the second lateral interface, encompassing TM-A, TM9 and TM12 on each of two symmetry-related mates. Direct end-to-end interactions between layers are mediated by a single though tight interface formed by TM-A, loop TM6–TM-A on the cytoplasmic side, and TM9 and loop TM9–TM10 on the periplasmic side.
(DOCX)

S2 Fig Expanded *B-*factor analysis on PepTs from various organisms. *(a)* P3_2_ crystal form of PepT_So_ from *Shewanella oneidensis* (PDB: 2XUT). Shown in a putty tube representation where the diameter of the tube is correlated with the magnitude of the C-alpha *B-*factors, and also colored by C-alpha *B-*factors: colors are ramped from blue over white to red, with blue designating low values and red designating high values. Two views are shown: cytoplasmic (top) and periplasmic (bottom). *(b)* P4_1_2_1_2 crystal form of PepT_So_ (PDB: 4UVM). *(c)* PepT_So2_ –a different PepT from *Shewanella oneidensis* (PDB: 4LEP). *(d)* GkPOT from *Geobacillus kaustophilus* (PDB: 4IKV). *(e)* YbgH from *Escherichia coli* (PDB: 4Q65). *(f)* YePEPT from *Yersinia enterolitica* (PDB: 4W6V). In the case of the P3_2_ crystal form of PepT_So_, most of the C-terminal MFS domain (TM7–TM12) as well as TM-A and TM-B are characterized by very high *B-*factors. The P4_1_2_1_2 form of this protein is structurally fairly similar, but here the *B-*factors are not as high overall. Nonetheless, the highest *B-*factors are still mainly in the C-terminal domain. In PepT_So2_, most of the high C-alpha *B-*factors are on the cytoplasmic side of the C-terminal MFS domain, but also the cytoplasmic side of the N-terminal MFS domain (TM1–TM6) features regions with high *B-*factors. In GkPOT, only TM-A and two loops on the periplasmic side of the C-terminal domain feature stretches with high C-alpha *B-*factors. In YbgH, regions with high *B-*factors are rather few and mainly include a few loops found on the cytoplasmic side of the N-terminal domain. In YePEPT, regions with high *B-*factors are even fewer, with loop TM1–TM2 sticking out the most. We conclude that the distributions of *B-*factors in the different PepTs are rather varied, and do not fully echo the scenario observed for PepT_St_.
(DOCX)
