# Supplementary material for: Structure determination of a major facilitator peptide transporter: Inward facing PepTSt from Streptococcus thermophilus crystallized in space group P3121
Source: PLoS One. 2017 Mar 6;12(3):e0173126. doi: 10.1371/journal.pone.0173126 (PMC5338821; doi:10.1371/journal.pone.0173126)
Supplement: S1 Fig — (a) The P3121 structure. Coloration is by residue count with colors ramped from white (N-terminus) over wheat, golden and orange to red (C-terminus). Side chains within 4 Å of a symmetry related molecule are shown in sticks with transparent surfaces, and the structural elements in which they occur are labeled. Two side views are shown. The contact areas on the periplasmic side are much more extensive than on the cytoplasmic side. Specifically, the former involves loop TM1–TM2, loop TM5–TM6, loop TM7–TM8, loop TM9–TM10 and loop TM11–TM12, and the latter TM7 and loop TM4–TM5. (b) The P212121 structure (PDB: 4APS). Shown as in panel a, except that resides within 4 Å of the other molecule in the asymmetric unit are shown in light blue sticks with transparent surfaces. The contact surfaces encompass fewer residues here, and they are more evenly distributed between the periplasmic and cytoplasmic sides. Specifically, they are found in loop TM1–TM2 and loop TM11–TM12 on the former side and loop TM6–TM-A on the latter (c). The C2221 form (PDB: 4D2C). Shown as in panel a. Two lateral interfaces are found. One is formed by the N-terminal tail, TM1, TM5, TM6, TM8 and loop TM7–TM8 packing against the equivalent elements of a symmetry-related mate to generate an antiparallel dimer, which is structurally equivalent to the non-crystallographic dimer seen in the P212121 form. These dimers are in turn linked into a continuous planar layers (Fig 4c, bottom) by the second lateral interface, encompassing TM-A, TM9 and TM12 on each of two symmetry-related mates. Direct end-to-end interactions between layers are mediated by a single though tight interface formed by TM-A, loop TM6–TM-A on the cytoplasmic side, and TM9 and loop TM9–TM10 on the periplasmic side. (DOCX) [file pone.0173126.s001.docx]

**Supporting information**

**
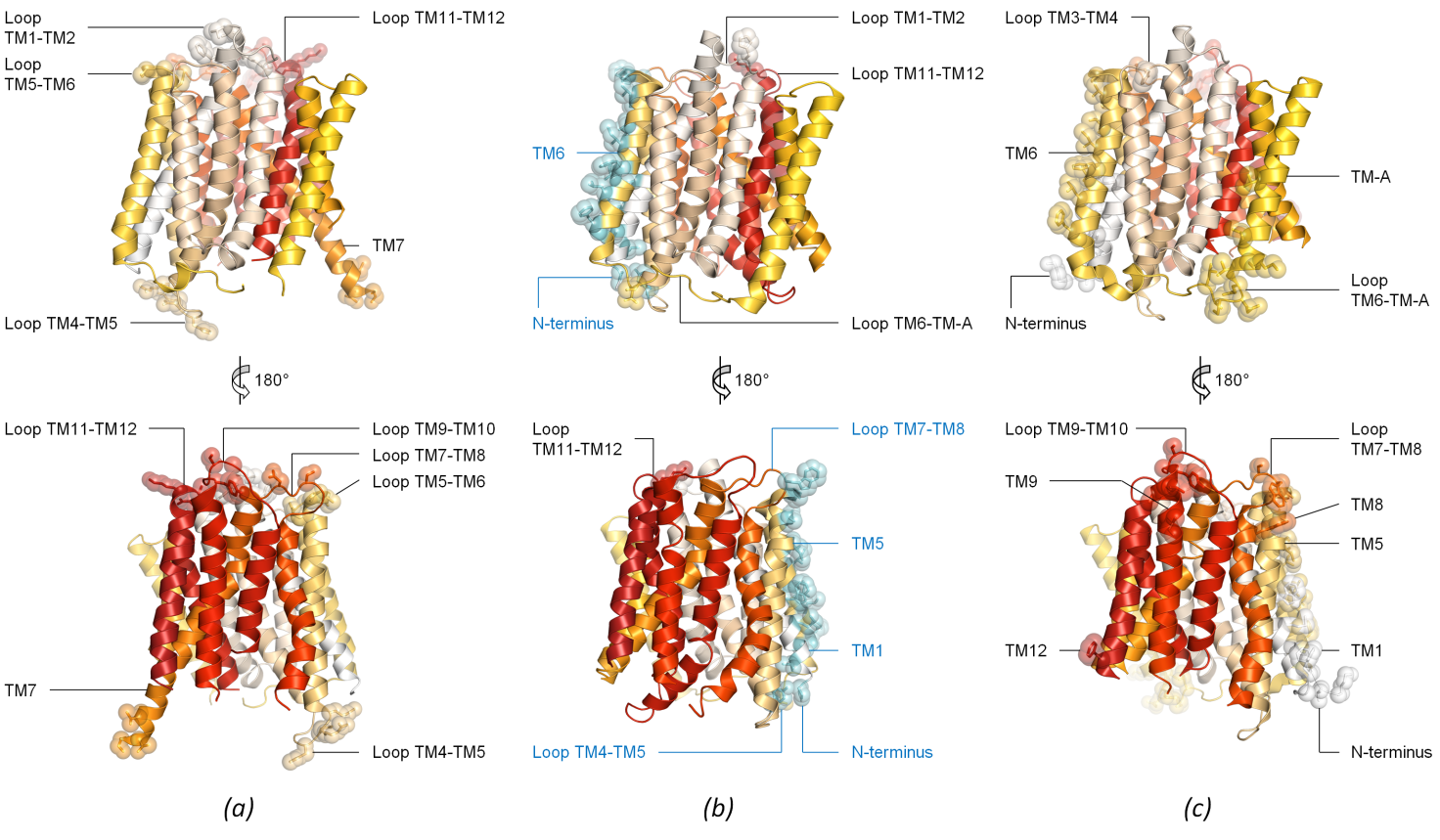
**

**S1 Fig. Crystal packing interfaces.**

*(a)* The P3_1_21 structure. Coloration is by residue count with colors ramped from white (N-terminus) over wheat, golden and orange to red (C-terminus). Side chains within 4 Å of a symmetry related molecule are shown in sticks with transparent surfaces, and the structural elements in which they occur are labeled. Two side views are shown. The contact areas on the periplasmic side are much more extensive than on the cytoplasmic side. Specifically, the former involves loop TM1–TM2, loop TM5–TM6, loop TM7–TM8, loop TM9–TM10 and loop TM11–TM12, and the latter TM7 and loop TM4–TM5. *(b)* The P2_1_2_1_2_1_ structure (PDB: 4APS). Shown as in panel *a*, except that resides within 4 Å of the other molecule in the asymmetric unit are shown in light blue sticks with transparent surfaces. The contact surfaces encompass fewer residues here, and they are more evenly distributed between the periplasmic and cytoplasmic sides. Specifically, they are found in loop TM1–TM2 and loop TM11–TM12 on the former side and loop TM6–TM-A on the latter *(c)*. The C222_1_ form (PDB: 4D2C). Shown as in panel *a*. Two lateral interfaces are found. One is formed by the N-terminal tail, TM1, TM5, TM6, TM8 and loop TM7–TM8 packing against the equivalent elements of a symmetry-related mate to generate an antiparallel dimer, which is structurally equivalent to the non-crystallographic dimer seen in the P2_1_2_1_2_1_ form. These dimers are in turn linked into a continuous planar layers (Fig. 4c, bottom) by the second lateral interface, encompassing TM-A, TM9 and TM12 on each of two symmetry-related mates. Direct end-to-end interactions between layers are mediated by a single though tight interface formed by TM-A, loop TM6–TM-A on the cytoplasmic side, and TM9 and loop TM9–TM10 on the periplasmic side.
