# Supplementary material for: Structure determination of a major facilitator peptide transporter: Inward facing PepTSt from Streptococcus thermophilus crystallized in space group P3121
Source: PLoS One. 2017 Mar 6;12(3):e0173126. doi: 10.1371/journal.pone.0173126 (PMC5338821; doi:10.1371/journal.pone.0173126)
Supplement: S2 Fig — (a) P32 crystal form of PepTSo from Shewanella oneidensis (PDB: 2XUT). Shown in a putty tube representation where the diameter of the tube is correlated with the magnitude of the C-alpha B-factors, and also colored by C-alpha B-factors: colors are ramped from blue over white to red, with blue designating low values and red designating high values. Two views are shown: cytoplasmic (top) and periplasmic (bottom). (b) P41212 crystal form of PepTSo (PDB: 4UVM). (c) PepTSo2 –a different PepT from Shewanella oneidensis (PDB: 4LEP). (d) GkPOT from Geobacillus kaustophilus (PDB: 4IKV). (e) YbgH from Escherichia coli (PDB: 4Q65). (f) YePEPT from Yersinia enterolitica (PDB: 4W6V). In the case of the P32 crystal form of PepTSo, most of the C-terminal MFS domain (TM7–TM12) as well as TM-A and TM-B are characterized by very high B-factors. The P41212 form of this protein is structurally fairly similar, but here the B-factors are not as high overall. Nonetheless, the highest B-factors are still mainly in the C-terminal domain. In PepTSo2, most of the high C-alpha B-factors are on the cytoplasmic side of the C-terminal MFS domain, but also the cytoplasmic side of the N-terminal MFS domain (TM1–TM6) features regions with high B-factors. In GkPOT, only TM-A and two loops on the periplasmic side of the C-terminal domain feature stretches with high C-alpha B-factors. In YbgH, regions with high B-factors are rather few and mainly include a few loops found on the cytoplasmic side of the N-terminal domain. In YePEPT, regions with high B-factors are even fewer, with loop TM1–TM2 sticking out the most. We conclude that the distributions of B-factors in the different PepTs are rather varied, and do not fully echo the scenario observed for PepTSt. (DOCX) [file pone.0173126.s002.docx]

**Supporting information**


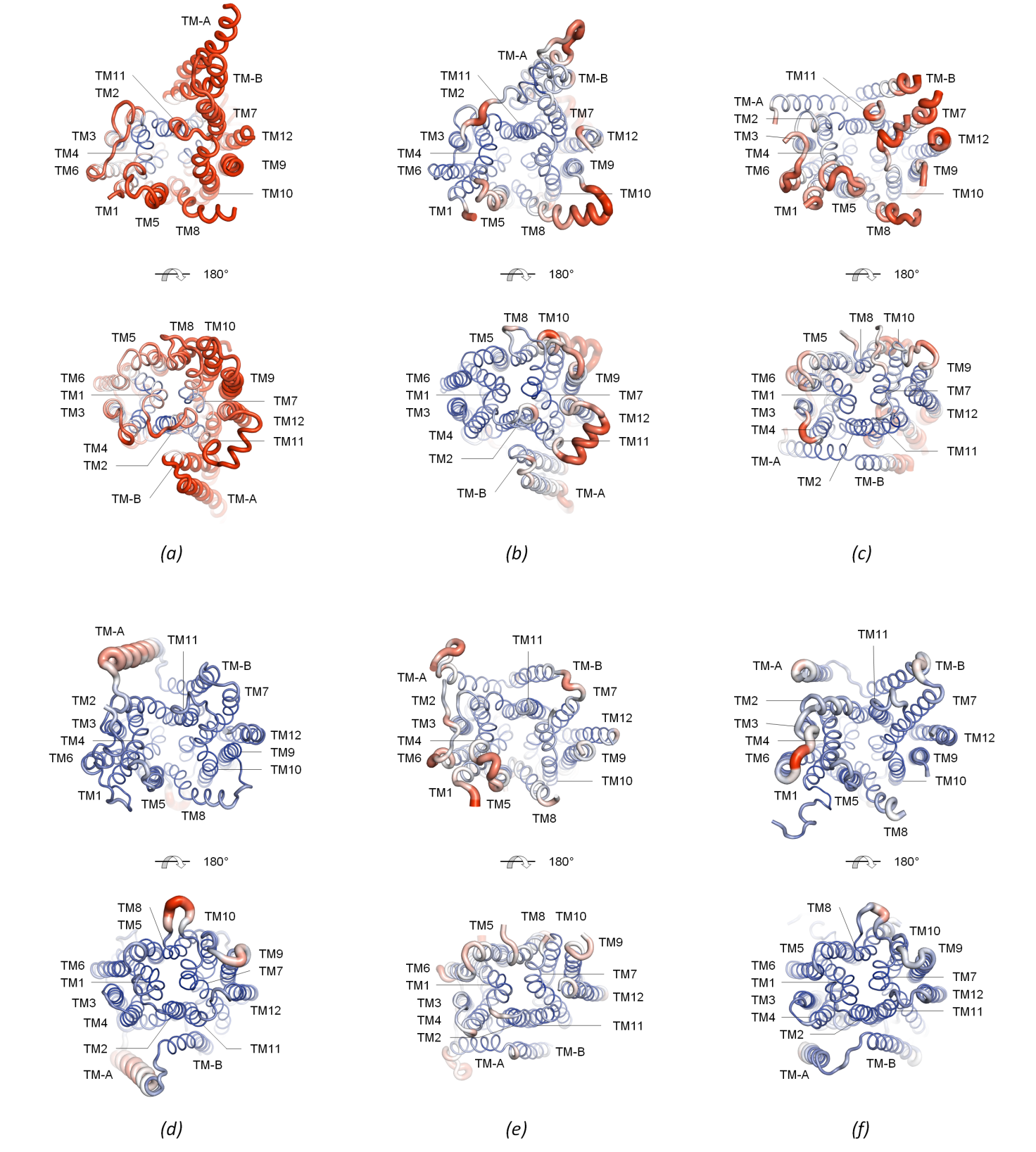


**S2 Fig. Expanded *B-*factor analysis on PepTs from various organisms.**

*(a)* P3_2_ crystal form of PepT_So_ from *Shewanella oneidensis* (PDB: 2XUT). Shown in a putty tube representation where the diameter of the tube is correlated with the magnitude of the C-alpha *B-*factors, and also colored by C-alpha *B-*factors: colors are ramped from blue over white to red, with blue designating low values and red designating high values. Two views are shown: cytoplasmic (top) and periplasmic (bottom). *(b)* P4_1_2_1_2 crystal form of PepT_So_ (PDB: 4UVM). *(c)* PepT_So2_ – a different PepT from *Shewanella oneidensis* (PDB: 4LEP). *(d)* GkPOT from *Geobacillus kaustophilus* (PDB: 4IKV). *(e)* YbgH from *Escherichia coli* (PDB: 4Q65). *(f)* YePEPT from *Yersinia enterolitica* (PDB: 4W6V). In the case of the P3_2_ crystal form of PepT_So_, most of the C-terminal MFS domain (TM7–TM12) as well as TM-A and TM-B are characterized by very high *B-*factors. The P4_1_2_1_2 form of this protein is structurally fairly similar, but here the *B-*factors are not as high overall. Nonetheless, the highest *B-*factors are still mainly in the C-terminal domain. In PepT_So2_, most of the high C-alpha *B-*factors are on the cytoplasmic side of the C-terminal MFS domain, but also the cytoplasmic side of the N-terminal MFS domain (TM1–TM6) features regions with high *B-*factors. In GkPOT, only TM-A and two loops on the periplasmic side of the C-terminal domain feature stretches with high C-alpha *B-*factors. In YbgH, regions with high *B-*factors are rather few and mainly include a few loops found on the cytoplasmic side of the N-terminal domain. In YePEPT, regions with high *B-*factors are even fewer, with loop TM1–TM2 sticking out the most. We conclude that the distributions of *B-*factors in the different PepTs are rather varied, and do not fully echo the scenario observed for PepT_St_.
